# Expanding the Therapeutic Landscape of Pericarditis: A Systematic Review of the Use of Conventional Immunosuppressants

**DOI:** 10.3390/medicina62050887

**Published:** 2026-05-05

**Authors:** Andrea Silvio Giordani, Caterina Menghi, Antonella Risoli, Anna Baritussio, Federico Scognamiglio, Matteo Castegnaro, Elena Pontara, Maria Grazia Cattini, Elisa Bison, Celeste Ambra Murace, Elena Verrecchia, Marco Giuseppe Del Buono, Francesco Landi, Ludovico Luca Sicignano, Alida Linda Patrizia Caforio

**Affiliations:** 1 Cardiology, Department of Cardiac Thoracic Vascular Sciences and Public Health, University of Padova, 35128 Padua, Italy; 2 Department of Geriatrics, Orthopaedics and Rheumatology, Università Cattolica del Sacro Cuore, Largo F. Vito 1, Fondazione Policlinico Universitario A. Gemelli IRCCS, 00168 Rome, Italyludovicoluca.sicignano@policlinicogemelli.it (L.L.S.); 3 Department of Medical and Surgical Sciences, Università Cattolica Sacro Cuore, Fondazione Policlinico Universitario A. Gemelli IRCCS, 00168 Rome, Italy; 4 Department of Cardiovascular Science, Università Cattolica Sacro Cuore, Fondazione Policlinico Universitario A. Gemelli IRCCS, 00168 Rome, Italy

**Keywords:** pericarditis, immunosuppressive therapy, idiopathic recurrent acute pericarditis, steroid-sparing therapy

## Abstract

***Background and Objectives***: While interleukin-1 inhibitors represent the standard of care for refractory idiopathic recurrent acute pericarditis, current guidelines also endorse conventional immunosuppressive (IS) agents as potential alternatives. The use of conventional IS agents is particularly relevant in specific clinical scenarios, such as systemic immune-mediated disease (SID)-associated pericarditis. However, existing evidence regarding their efficacy and safety for pericarditis treatment remains fragmented, deriving exclusively from case reports, case series, and small monocentric observational studies. Our aims are: To characterize the clinical and diagnostic profiles of patients with pericarditis treated with conventional IS agents and to evaluate the therapeutic efficacy and safety of such agents. ***Materials and Methods***: A systematic review was conducted in accordance with PRISMA guidelines. Major electronic databases were searched from January 1970 to March 2026 for case reports, case series, and observational studies detailing the use of conventional IS therapies for pericarditis. Clinical and therapeutic data, including specific IS indications and dosing regimens, were systematically extracted. ***Results***: The final analysis included 39 reports comprising 75 patients (60% female; median age 36.0 years). The underlying pericarditis aetiology was predominantly SID-related (53%, n = 40) or idiopathic/presumed viral recurrent disease (40%, n = 30). The most frequently prescribed first-line IS agents were azathioprine (44%) and methotrexate (25%). Across published reports, IS therapy was described as achieving pericarditis clinical resolution in all cases and facilitated corticosteroid withdrawal in 72% of patients. Overall, pericarditis recurrence while on IS therapy occurred in only 10% of the cohort. Adverse events requiring IS withdrawal were rare (n = 2, 3%). ***Conclusions***: Conventional IS agents appear effective and generally well tolerated in the published literature on SID-associated and isolated recurrent pericarditis. These findings reinforce the clinical utility of conventional IS therapies as a viable, steroid-sparing strategy when targeted biologic therapies lack sufficient investigation.

## 1. Introduction

Idiopathic recurrent acute pericarditis (IRAP) represents a challenging clinical entity, affecting up to 30% of patients with acute pericarditis and accounting for a disproportionate burden of morbidity, healthcare utilization, and impaired quality of life [1]. Although interleukin-1 (IL-1) inhibition has emerged as the standard of care for colchicine-resistant and/or corticosteroid-dependent forms, real-world practice may encounter clinical scenarios in which biologic therapy is not the primary indication or insufficiently studied, such as IRAP in the context of systemic immune-mediated diseases (SIDs) [2,3,4,5]. In these settings, conventional immunosuppressive (IS) agents—long used empirically before the advent of targeted therapies—retain potential therapeutic value [6]. Despite decades of clinical experience, the evidence base supporting these agents remains fragmented, largely limited to small cohorts, sparse case reports and small monocentric observational studies [7]. Agents such as azathioprine (AZA), methotrexate (MTX), mycophenolate mofetil (MMF), cyclophosphamide (Cyc), and intravenous immunoglobulins (IVIG) have been used in selected patients, often guided by comorbid SID, steroid toxicity, or lack of response to first and/or second line agents. Notably, early observational experiences suggested meaningful steroid-sparing effects and durable remission in selected subsets of patients. Given the evolving therapeutic landscape, a comprehensive synthesis of the role, efficacy, and safety of conventional immunosuppressants is warranted.

This comprehensive review aims to contextualize the use of conventional IS agents within modern pericarditis management, delineate clinical scenarios where they may remain relevant, and highlight knowledge gaps requiring prospective research.

## 2. Methods

### 2.1. Study Design and Search Strategy

The systematic review of the literature was undertaken according to the PRISMA (Preferred Reporting Items for Systematic reviews and Meta-Analysis) guidelines for its design, implementation, analysis, and reporting [8] (Supplementary Table S1). A comprehensive literature search was performed across EMBASE, PubMed (MEDLINE), and Web of Science from January 1970 to 10 March 2026. The search strategy combined free-text terms and Medical Subject Headings (MeSH) related to pericarditis and conventional IS agents (details in: Supplementary Materials Section S1). Furthermore, the reference lists of the 2015 and 2025 European Society of Cardiology (ESC) Guidelines on the management of pericardial diseases, as well as all studies previously identified as having met the inclusion criteria, were manually reviewed for additional relevant publications.

Given the nature of the available evidence, true efficacy rates could not be estimated, due to the likely relevant publication bias intrinsic to case reports and small case series, which preferentially describe successful therapeutic experiences.

### 2.2. Eligibility Criteria

We included case reports, case series, and observational studies reporting clinical and therapeutic data on the use of conventional IS agents (e.g., AZA, MTX, MMF, Cyc) for the treatment of pericarditis. Aggregated data from clinical trials were not considered, in order to ensure methodological consistency with an individual patient data (IPD) approach. Reports detailing cases of serositis without clear pericardial involvement, as well as cases of isolated pericardial effusion without a confirmed diagnosis of pericarditis, were excluded.

### 2.3. Data Extraction and Management

**Figure 1:**
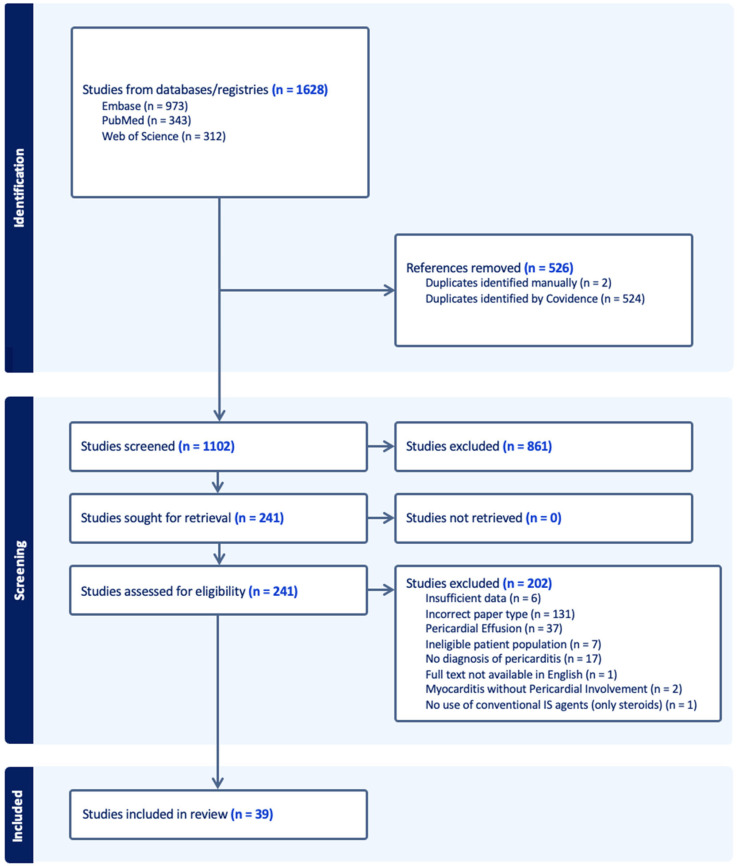
PRISMA flow diagram: literature search and study selection process.

All retrieved citations were imported into Covidence systematic review software (*Veritas Health Innovation*, *Melbourne*, *Australia*) for management and deduplication. Following the removal of 526 duplicates (524 automatically identified by Covidence and 2 manually), a total of 1102 records were screened (Figure 1). Two independent authors (A.S.G. and C.M.) performed the analysis of the titles and abstracts. Subsequently, 861 studies were excluded, and 241 full texts were assessed for final eligibility.

Following full-text evaluation, 202 records were excluded for the following reasons: incorrect paper type (e.g., narrative review, n = 131), pericardial effusion without pericarditis (n = 37), unconfirmed diagnosis of pericarditis (n = 17), ineligible patient population (e.g., no conventional IS used, n = 7), insufficient data (n = 6), myocarditis without pericardial involvement (n = 2), and no use of conventional IS agents (n = 1). Ultimately, 39 reports were included in the final analysis [9,10,11,12,13,14,15,16,17,18,19,20,21,22,23,24,25,26,27,28,29,30,31,32,33,34,35,36,37,38,39,40,41,42,43,44,45,46,47]. Discrepancies between reviewers were resolved by consensus or by a third senior author (A.R.), if necessary.

Clinical, laboratory, imaging, and therapeutic data were extracted from article texts, tables, and figures. Follow-up time was considered as the longest reported. The final dataset predominantly consisted of individual patient data (IPD) derived from case reports and case series, with the inclusion of one retrospective cohort study that provided exclusively aggregated data [17].

Pericarditis resolution was defined as disappearance of the clinical and/or diagnostic features that supported pericarditis diagnosis, specifically chest pain, ECG changes, pericardial effusion and/or friction rubs. When concurrent myocarditis was reported, the diagnosis was accepted if based on established clinical guidelines [1], specifically requiring the presence of characteristic findings on Cardiac Magnetic Resonance (CMR) imaging and/or elevated cardiac troponin.

### 2.4. Quality Assessment

The methodological quality of the included case reports and case series was independently evaluated by two reviewers using the Murad tool for assessing the methodological quality of case reports and case series [48,49].

### 2.5. Risk of Bias Assessment

Given the absence of validated tools for the type of evidence included in our study, the risk of bias was assessed using an adapted framework for case reports and case series. Two reviewers (A.S.G. and C.M.) independently evaluated each report with respect to clarity of case selection and inclusion criteria; adequacy of diagnostic criteria for pericarditis; consistency of clinical, laboratory, and imaging data; details of therapeutic interventions; clarity of outcome reporting. Since our review only included uncontrolled case-based studies, the risk of bias assessment was qualitative, intending to contextualize the reliability of the extracted data.

### 2.6. Statistical Analysis

Summary statistics of variables were expressed as median and interquartile range (Q1–Q3), or absolute number and percentage, as appropriate. Continuous variables were compared using the Mann-Whitney U test; categorical variables were compared using Pearson’s Chi-squared tests or Fisher’s exact tests, depending on the expected frequencies. Categorical variables from the single aggregated study were pooled with the individual-level data to calculate overall frequencies and proportions. Conversely, for continuous numerical variables (specifically time to first recurrence, median oral steroid daily dose, months of IS therapy, duration of follow-up, and months to recurrence on IS), direct IPD pooling was not feasible without the original raw data. Therefore, summary statistics for these continuous variables from the aggregated cohort were not merged but were maintained and reported separately as medians and interquartile ranges (Q1, Q3) within the descriptive tables. Statistical significance was based on a two-sided *p*-value threshold of <0.05. To account for multiple comparisons, *p*-values were adjusted using the Benjamini-Hochberg False Discovery Rate (FDR) procedure. The analyses were performed using Jamovi software (version 2.7). Considering the small sample size, the heterogeneity of clinical contexts, and the inclusion of case reports and case series, the statistical comparisons were exploratory and intended to describe patterns within the published data, with no possibility to support inferential conclusions.

## 3. Results

### 3.1. Characteristics of the Included Studies

The 39 included studies yielded data on 75 patients, with reports published between 1970 and 2026 (Table 1). The median age at diagnosis was 36.0 years (Q1–Q3, 23.0–50.0), with a female predominance (n = 37, 60%). The cohort was predominantly of Caucasian descent (n = 46, 81%), alongside patients of African (n = 6, 11%), Asian (n = 4, 7%), and Hispanic (n = 1, 2%) ethnicities.

SIDs were the most common underlying etiology (n = 40, 53%), followed by idiopathic/presumed viral pericarditis (n = 30, 40%). Less frequent causes included iatrogenic pericardial injury (n = 3), post-myocardial infarction syndrome (n = 1), and immune-checkpoint inhibitor (ICI)-associated pericarditis (n = 1). Among the 40 SID-related cases, systemic lupus erythematosus (SLE) was the most frequent condition (n = 23), followed by rheumatoid arthritis (n = 7) and eosinophilic granulomatosis with polyangiitis (n = 2). The remaining SID cases (n = 8) were attributed to rarer conditions, including adult-onset Still’s disease (n = 2), Behçet’s disease (n = 1), Chronic Atypical Neutrophilic Dermatosis with Lipodystrophy and Elevated temperature (CANDLE) syndrome (n = 1), Crohn’s disease (n = 1), polymyositis (n = 1), sarcoidosis (n = 1), and granulomatosis with polyangiitis (n = 1).

At the index hospitalization, 66% of patients had a history of recurrent pericarditis, with the index flare occurring relatively soon after the previous episode (median time to recurrence: 3.0 months). Pleuritic chest pain was the hallmark symptom (n = 50, 81%), frequently accompanied by fever (n = 30, 57%) and pericardial friction rubs (n = 17, 33%). Electrocardiography revealed widespread ST-segment elevation and/or PR-segment depression, suggestive of acute pericarditis, in 58% of evaluated cases (n = 26). Systemic in€flammation was prominent, with C-Reactive Protein (CRP) elevated in 91% of tested patients (n = 61; median peak: 69.0 mg/L). Concomitant myocardial involvement (i.e., associated myocarditis) was diagnosed in 20% of the cohort (n = 10), corroborated by elevated troponin levels in 8 cases.

Echocardiography identified pericardial effusion in 89% of patients (n = 42), which was graded as mild (n = 7), moderate (n = 9), or severe (n = 11). Left ventricular ejection fraction (LVEF) was generally preserved (n = 35, 95%). CMR imaging was performed in 12 cases; of these, 8 showed signs of myocardial and/or pericardial inflammation.

Cardiac tamponade complicated the clinical course in 13 cases (22%), mandating pericardiocentesis in all instances; pericardial fluid analysis did not lead to specific etiological diagnosis in any of the reported cases. Signs of pericardial constriction at diagnosis were observed in 5 patients (8%), leading to surgical pericardiectomy in 3 cases.

### 3.2. Medical Therapy for Pericarditis: First-Line Therapy and Rationale for Immunosuppression

Prior to the initiation of conventional IS, standard-of-care regimens included corticosteroids in 87% of patients (intravenous in 5 cases), NSAIDs in 41% (n = 31), and colchicine in 35% (n = 26) (Table 1). 

The primary clinical indications for initiating conventional IS were: refractory pericarditis (n = 39, 52%), exacerbation of the underlying systemic disease (n = 20, 27%), and corticosteroid dependence (n = 16, 21%, i.e., used as a steroid-sparing strategy in the absence of systemic involvement). The most frequently prescribed first-line agents were AZA (n = 33, 44%), MTX (n = 19, 25%), MMF (n = 11, 15%), and Cyc (n = 8, 11%). After failure of first-line medical treatment, a second-line IS agent was required in 13 cases, with 4 patients eventually progressing to a third-line agent and 1 patient to a fourth-line therapy.

In all reported cases, IS therapy resulted in clinical resolution of pericarditis. Furthermore, among patients receiving baseline corticosteroids, the introduction of IS enabled steroid withdrawal in 72% of cases (n = 42). During follow-up, pericarditis recurrence while on active IS therapy occurred in only 10% of patients (n = 6), with a median time to relapse of 2.0 months.

The safety profile was favourable, with only 2 cases of reported adverse events (2.6%) that necessitated treatment discontinuation in both instances (infection on MTX, n = 1 [9]; pancytopenia on AZA, n = 1 [37]).

### 3.3. IS Treatment for Recurrent Pericarditis

We compared data of patients treated with IS at their first pericarditis episode (n = 25) and patients treated with IS at pericarditis relapse (n = 49) (Table 2). Patients with a history of previous pericarditis events showed a more pronounced inflammatory clinical presentation, with significantly higher frequencies of pericarditic chest pain (97% vs. 56%, *p* < 0.001), friction rubs (52% vs. 8.3%, *p* < 0.001), and typical ECG alterations (77% vs. 32%, *p* = 0.002). Interestingly, all patients (100%) in the recurrent group had elevated CRP, compared to 71% of patients treated at their first episode (*p* < 0.001). No significant differences were observed between the two groups regarding tamponade (*p* = 0.056) or constriction (*p* > 0.999) at diagnosis.

Patients treated with IS agents at their first pericarditis episode were exclusively affected by SIDs (100% vs. 29%, *p* < 0.001) and required a significantly higher median dose of oral steroids before IS initiation (60.0 mg/day vs. 20.0 mg/day, *p* = 0.005), whereas patients with previous events more frequently received colchicine (47% vs. 8.0%, *p* < 0.001) and NSAIDs (51% vs. 20%, *p* = 0.010).

Conventional IS indications also differed significantly (*p* < 0.001): first-event patients were primarily treated for SID flares (68% vs. 6.1%), whereas patients with pericarditis recurrence were mainly treated for refractory pericarditis (65% vs. 24%) or steroid dependence (29% vs. 8.0%). Finally, while the recurrent group had a higher success rate in withdrawing steroids (86% vs. 45%, *p* < 0.001), they also experienced a higher rate of recurrence while on IS therapy (36% vs. 4.0%, *p* = 0.008).

### 3.4. IS Treatment for SIDs-Related Pericarditis vs. Isolated Pericarditis

We compared data of patients with SIDs (n = 40) and without SIDs (n = 35) (Table 3). At diagnosis, SIDs patients presented with a less typical inflammatory pericarditic phenotype, showing significantly lower chest pain occurrence (70% vs. 100%, *p* = 0.005), friction rubs (16% vs. 73%, *p* < 0.001), and typical ECG alterations (45% vs. 86%, *p* = 0.011). Nevertheless, SIDs patients experienced significantly higher rates of cardiac tamponade (35% vs. 3.8%, *p* = 0.003) and more frequently required pericardiocentesis (32% vs. 7.7%, *p* = 0.022).

Regarding therapeutic management, all (100%) non-SIDs patients had a history of prior recurrences compared to 36% of SIDs patients (*p* < 0.001). Conventional IS indications also varied significantly between the two groups (*p* < 0.001): SIDs patients were predominantly treated for a systemic disease flare (50%) or refractory disease (45%), while non-SIDs patients were mostly treated for refractory disease (60%) or steroid dependence (40%). When analysing specific IS agents, MMF was exclusively used in SIDs patients (28% vs. 0%, *p* < 0.001), while AZA was more frequently prescribed in patients without SIDs (AZA: 69% vs. 23%; *p* < 0.001). Furthermore, the use of multiple lines of IS was more frequent in SID patients.

Consistent with the findings in the cohort with previous pericarditis episodes, non-SIDs patients showed a higher success rate in progressively withdrawing steroids (90% vs. 55%, *p* = 0.003) but also experienced a higher rate of pericarditis recurrence while on IS therapy (44% vs. 10%, *p* = 0.001).

A sensitivity analysis restricted to the 38 reports providing individual patient data (n = 62 patients) showed similar patterns of clinical baseline features and clinical response across all patient’s subgroups, supporting the robustness of the overall findings (Supplementary Tables S3–S5).

### 3.5. Quality Assessment

Based on the Murad tool, more than half of the studies (24/39, 62%) were rated as good quality (score ≥ 6), while the remaining 15 (15/39, 38%) were of intermediate quality (score 4–5) (Supplementary Table S2) [49].

## 4. Discussion

The main findings of our study are: first, IS therapy achieved clinical resolution of pericarditis in all reported cases and demonstrated a favourable safety profile, even in complex presentations complicated by cardiac tamponade or pericardial constriction. Despite likely influenced by reporting bias, these findings reinforce the clinical utility of conventional IS therapies, which can have a role even in the present time, in which anti-IL1 agents have become standard of care for recurrent pericarditis.

Second, clinicians primarily prescribed IS therapy for patients with concurrent SIDs, particularly SLE and RA. In this population, IS agents effectively controlled both pericardial involvement and underlying systemic disease, though some patients required multiple lines of therapy. Finally, in the subset of patients with isolated recurrent pericarditis, IS therapy was primarily indicated as a steroid-sparing strategy for steroid-dependent disease, with AZA and MTX being the most frequently chosen agents.

### 4.1. The Use of IS in the Modern Therapeutic Landscape of Pericarditis

Our systematic review shows that, despite the recent paradigm shift toward targeted IL-1 blockade [50], IS agents may remain clinically relevant across a broad spectrum of pericarditis presentations. This is particularly evident in the real-world scenarios captured by the 75 patients included in our analysis, in whom IS therapy achieved complete clinical resolution and enabled corticosteroid withdrawal in 72% of those receiving baseline steroids. Nevertheless, it should be acknowledged that diagnostic criteria and therapeutic strategies for pericarditis and associated conditions have evolved substantially during these decades. In particular, the introduction of colchicine in the early 2000s [51] and IL-1 inhibitors in the past decade [1] has reshaped the management of recurrent pericarditis, potentially influencing both treatment selection and clinical trajectories.

Notably, IS use was not confined to historical practice: although the reports span several decades (1970–2026), more than half of the cases (53%) involved pericarditis associated with SIDs, a setting in which IL-1 inhibitors remain insufficiently studied and may not adequately address multisystem inflammatory activity. In these patients, IS agents served a dual therapeutic purpose, controlling pericardial inflammation and treating the underlying disease.

Notably, 12 of the 39 included reports (31%) were published prior to the 2015 European Society of Cardiology guidelines [52], which standardized pericarditis management and recommended colchicine from the initial episode to reduce recurrence risk and improve disease control [51] (Supplementary Figure S1). This temporal heterogeneity likely contributed to variability in treatment strategies, including the timing, dosing, and duration of IS therapy. However, even in more contemporary cases, the optimal regimen and duration of IS treatment—particularly in isolated, non-SID associated pericarditis—remain poorly defined. This mirrors the broader uncertainty surrounding long-term medical therapy for idiopathic recurrent pericarditis, where prolonged treatment with IL-1 inhibitors is often required [53]. Prospective studies are needed to define optimal IS dosing strategies and treatment duration in pericarditis.

Moreover, despite IS agents having occasionally been implicated as potential causes of pericarditis—most notably MTX [54], but also AZA [55]—such events appear exceedingly rare. In our review, 33 patients were treated with AZA and 19 with MTX, with a favourable efficacy and safety profile and no reported cases of drug-induced pericarditis. Notably, while IL-1 blockade has demonstrated superior efficacy in randomized trials, with rapid symptom resolution, normalization of inflammatory markers, and markedly reduced recurrence rates compared with historical standards of care, the use of IS in pericarditis derives exclusively from case reports and small observational studies, limiting direct comparability, although many IS agents have decades of real-world experience in autoimmune disease management. Nevertheless, this review highlights the potential role of IS therapy in restricted clinical scenarios, specifically as a steroid-sparing alternative when biologic therapies are unavailable, contraindicated (e.g., due to anaphylaxis), or economically inaccessible.

### 4.2. Immunosuppressive Agents Across Heterogeneous Pericarditis Clinical Presentations

The role of conventional IS in pericarditis appears to vary substantially across different clinical phenotypes, particularly when comparing SID-associated forms with non-SID presentations. In the context of SID-associated pericarditis, pericardial involvement is generally considered to be a component of a more extensive systemic autoimmune process, as opposed to being an isolated serous manifestation. Pericardial disease is less prevalent, yet adequately documented in RA, with a prevalence of approximately 10–30% in echocardiographic studies, while clinically evident pericarditis is comparatively rare [2].

In this setting, conventional IS such as MMF are primarily utilised as steroid-sparing agents or as background therapies aimed at controlling the underlying disease activity. Consequently, the utilisation of these drugs is frequently driven more by the systemic disease framework than by pericardial inflammation per se, especially in conditions such as SLE or other connective tissue diseases. Conversely, non-SID pericarditis manifests more commonly with a distinctly inflammatory phenotype, characterised by elevated acute-phase reactants, the presence of typical chest pain, electrocardiographic changes, and, in certain instances, fever and serositis extending beyond the pericardium. This clinical profile overlaps with the so-called “autoinflammatory endotype”, which is primarily driven by interleukin-1–mediated pathways [56,57]. In this context, targeted therapies such as anakinra have demonstrated consistent efficacy, suggesting that conventional IS may be less biologically aligned with the dominant pathogenic mechanisms in these patients [58]. Although direct comparative evidence is lacking, these observations support a phenotype-driven therapeutic approach: conventional IS agents are likely less effective than IL-1 blockade for highly inflammatory, non-SID pericarditis.

In more complex clinical scenarios, such as constrictive physiology or cardiac tamponade, the role of conventional IS agents is more limited and context-dependent. In cases of cardiac tamponade, urgent pericardial drainage remains the cornerstone of management, with the consideration of immunosuppressive therapy only after haemodynamic stabilisation and an assessment of the underlying cause, particularly in cases linked to systemic autoimmune disease [52]. In constrictive pericarditis, particularly in its transient or inflammatory forms, immunomodulatory strategies have been shown to contribute to the reversal of inflammation-driven constriction. However, current evidence appears to offer stronger support for the utilisation of targeted anti-IL-1 therapies as opposed to conventional IS in this context [58,59]. Our findings underscore the significance of a phenotype-oriented approach to pericarditis management. Conventional IS agents appear to play a complementary and context-specific role, particularly in cases associated with SID and in cases with myocardial involvement. However, their utility in non-SID, highly inflammatory phenotypes and in acute complicated presentations remains more limited. As visually contextualized in our temporal distribution chart (Supplementary Figure S1), this continued reliance on conventional IS agents in recent years may suggest a potential delay in adopting guideline-recommended stepwise approaches, or difficulties in accessing targeted biologic therapies. Crucially, however, this trend is also largely driven by the high prevalence of SIDs in our cohort, for which IL-1 inhibitors do not currently represent the first-line therapeutic option [2,3,4,5].

### 4.3. Different Therapeutic Pathways in SID-Related vs. Isolated Recurrent Pericarditis

Our systematic review underscores distinct therapeutic strategies between SIDs-related pericarditis and isolated forms. In patients with SIDs, conventional IS agents were primarily initiated to control systemic disease activity rather than pericardial inflammation *per se*, although they ultimately led to resolution of pericarditis in all cases. This population frequently required a stepwise intensification strategy, escalating to multiple IS lines due to suboptimal control of the underlying autoimmune condition with first-line therapies. Notably, MMF was used exclusively in the SID cohort. In contrast, among non-SID patients, conventional IS (mainly AZA and MTX) were introduced specifically for the management of isolated, corticosteroid-dependent or refractory pericarditis, with the goal of achieving sustained remission and complete steroid withdrawal.

Consistent with these differing therapeutic aims, the non-SID cohort achieved a significantly higher rate of steroid independence compared with SID patients (90% vs. 55%). However, this apparent advantage was offset by a higher incidence of recurrences during ongoing IS therapy (44% vs. 10%). The challenge of maintaining durable remission in this population is consistent with prior single-center studies [6]. In a retrospective cohort of 46 patients with isolated recurrent pericarditis, AZA enabled sustained remission and corticosteroid discontinuation in over half of cases; nevertheless, approximately 37% of patients experienced relapse during steroid tapering.

Accurate etiological classification remains pivotal for treatment individualization, yet real-world data continue to highlight substantial gaps in guideline adherence. A recent nationwide registry [60] reported unexpectedly low colchicine utilization—17.0% in autoimmune-associated pericarditis and 23.2% in idiopathic cases—despite extensive corticosteroids use. Our review mirrors this trend: prior to the initiation of conventional IS, only 35% of patients had received colchicine and 41% NSAIDs (with no differences between SID-related and isolated pericarditis), while 87% were already corticosteroid-dependent. Thus, while identifying an underlying autoimmune aetiology is pivotal—given that these patients may exhibit a more favourable response to conventional IS therapy—the pharmacological management of pericarditis must still follow a structured stepwise approach: strict adherence to optimized treatment courses with NSAIDs and colchicine remains essential [1], in order to prevent recurrences and reduce the risk of corticosteroid dependence in both SID-related and isolated pericarditis.

## 5. Limitations

The main limitation of this review is the strong likelihood of publication bias, given that 100% of the included cases reported IS agents as effective in pericarditis control, with no documented instances of medical therapy failure.

Second, despite the dataset spans a large period of time (included reports publication date from 1970 to 2026), given the small number of cases per each time period, meaningful stratified analyses were not feasible.

In addition, hypothesis testing in our study is limited by the small sample size, the heterogeneity of the data, and the case-based nature of included reports; thus, all statistical comparisons are exploratory and purely descriptive.

Another relevant limitation is the nature of the available evidence, which consists exclusively of case reports and small case series with heterogeneous reporting quality; as a result, some clinically relevant variables (such as friction rubs, standardized treatment protocols) may have been inconsistently documented. Notably, the overall utilization of CMR imaging was low, even in patient subgroups with complicated disease courses—such as those with associated myocarditis or constrictive pericarditis—where modern guidelines would indicate its use. This underutilization likely reflects the extended publication time span of the included reports.

Treatment regimens varied widely across decades, leading to high heterogeneity of treatment types and durations; for example, colchicine was approved for recurrent pericarditis treatment after the publication of many of the reports.

Finally, the absence of controlled studies prevents firm conclusions regarding comparative efficacy, optimal dosing, and treatment duration.

## 6. Conclusions

Conventional IS agents appear to be generally effective and safe for the management of pericarditis, including cases associated with SIDs and isolated recurrent idiopathic forms. These findings support the use of conventional IS for selected cases in which anti-IL1 therapies do not represent the primary indication (e.g., SIDs), or are insufficiently studied, while highlighting the need for prospective studies to define optimal regimens and treatment duration.

## Figures and Tables

**Table 1:** Baseline Demographics, Clinical Characteristics, and Treatment Outcomes of the Overall Cohort.

Characteristic	Overall Cohort (N = 75) ^1^	Missing (n)
*Demographics*		
Age, years	36.0 (23.0, 50.0)	13
Female Sex	37/62 (60%)	13
Ethnicity		18
African	6/57 (11%)	
Asian	4/57 (7.0%)	
Caucasian	46/57 (81%)	
Hispanic	1/57 (1.8%)	
Pericarditis Etiology		0
SIDS (Autoimmune)	40/75 (53%)	
Idiopathic (presumed viral)	30/75 (40%)	
Iatrogenic pericardial injury	3/75 (4.0%)	
Post-myocardial infarction (Dressler Syndrome)	1/75 (1.3%)	
ICI-associated pericarditis	1/75 (1.3%)	
Autoimmune Disease	40/75 (53%)	0
Underlying Autoimmune Disease		
SLE	23/42 (55%)	
RA	7/42 (17%)	
Systemic Sclerosis	2/42 (4.8%)	
EGPA	2/42 (4.8%)	
Other *	8/42 (19%)	
*Clinical Presentation*		
Prior Recurrences	49/74 (66%)	1
Months Since Previous Episode		
Individual Data (n = 22)	3.0 (1.0, 7.0)	14
Aggregated Data (n = 13)	17.0	
Chest Pain	50/62 (81%)	13
Friction Rub	17/52 (33%)	23
Fever	30/53 (57%)	22
ECG suggestive of acute pericarditis	26/45 (58%)	30
Associated myocarditis	10/49 (20%)	26
Pericardial constriction at Diagnosis	5/60 (8.3%)	15
Pericardiectomy	3/60 (5.0%)	15
*Laboratory & Imaging Findings*		
Elevated CRP	61/67 (91%)	8
CRP Peak Value, mg/L	69.0 (12.0, 146.0)	43
Abnormal Troponin	8/41 (20%)	34
LVEF by Echo		38
Preserved	35/37 (95%)	
Mildly Reduced	1/37 (2.7%)	
Moderately Reduced	1/37 (2.7%)	
Pericardial Effusion (present)	42/47 (89%)	28
Pericardial Effusion Size		15
Mild	7/27 (26%)	
Moderate	9/27 (33%)	
Severe	11/27 (41%)	
Cardiac Tamponade	13/60 (22%)	15
Pericardiocentesis	13/60 (22%)	15
CMR Performed	12/46 (26%)	29
*Pericarditis medical therapy*		
Prior NSAID	31/75 (41%)	0
Prior Colchicine	26/75 (35%)	0
Prior Steroid	65/75 (87%)	0
IV Steroid Dose Pre-IS, mg/day	60.0 (60.0, 250.0)	
Oral Steroid Dose Pre-IS, mg/day		
Individual Data (n = 35)	20.0 (20.0, 60.0)	
Aggregated Data (n = 13)	20.0	
IS therapy Indication		0
Refractory disease	39/75 (52%)	
Steroid-dependence	16/75 (21%)	
Systemic disease flare	20/75 (27%)	
First IS regimen		
AZA	33/75 (44%)	
MTX	19/75 (25%)	
MMF	11/75 (15%)	
Cyc	8/75 (11%)	
Other IS regimens ^	4/75 (5.3%)	
IS Duration, months		23
Individual Data (n = 39)	6.0 (3.0, 12.0)	
Aggregated Data (n = 13)	6.0	
Steroid Withdrawal	42/58 (72%)	17
Follow-up, months		
Individual Data (n = 39)	7.0 (6.0, 18.0)	12
Aggregated Data (n = 13)	6.0	
*Outcomes*		
Pericarditis Resolved	70/70 (100%)	5
Recurrence on Conventional IS	6/60 (10%)	15
Months to Recurrence	2.0 (1.5–2.0)	3
Adverse Event	2/75 (2.6%)	0
IS Stopped for AE	2/75 (2.6%)	0

^1^ Median (Q1, Q3); n/N (%). AE: adverse event; AZA: azathioprine; CMR: cardiovascular magnetic resonance; CRP: C-reactive protein; Cyc: cyclophosphamide; ECG: electrocardiogram; EGPA: eosinophilic granulomatosis with polyangiitis; ICI: immune checkpoint inhibitor; IS: immunosuppression; IV: intravenous; LVEF: left ventricular ejection fraction; MMF: mycophenolate mofetil; MTX: methotrexate; NSAID: non-steroidal anti-inflammatory drug; RA: rheumatoid arthritis; SIDS: systemic immune-mediated diseases; SLE: systemic lupus erythematosus. * Other specific autoimmune diseases include: Adult-onset Still’s disease (n = 2, 4.8%), Behcet’s disease (n = 1, 2.4%), CANDLE (n = 1, 2.4%), Crohn’s disease (n = 1, 2.4%), Polymyositis (n = 1, 2.4%), Sarcoidosis (n = 1, 2.4%), and Granulomatosis with polyangiitis (GPA) (n = 1, 2.4%). ^ The “Other IS” category includes: Cyclosporine A (n = 1, 1.3%), Etanercept (n = 1, 1.3%), Hydroxychloroquine (n = 1, 1.3%) and Infliximab (n = 1, 1.3%).

**Table 2:** Baseline Characteristics, Therapeutic Management, and Clinical Outcomes Stratified by First-Episode versus Recurrent Pericarditis.

Characteristic	Overall (n = 74) ^1^	First Event (n = 25) ^1^	Recurrence (n = 49) ^1^	*p*-Value ^2^	q-Value ^3^	Missing (n)
Demographics						
Age, years	35.0 (23.0, 49.0)	34.0 (23.0, 53.0)	37.0 (22.5, 48.0)	0.613	0.840	13
Female Sex	36/61 (59%)	12/25 (48%)	24/36 (67%)	0.145	0.268	13
Ethnicity				**0.003**	**0.008**	18
Caucasian	45/56 (80%)	12/20 (60%)	33/36 (92%)			
African	6/56 (11%)	4/20 (20%)	2/36 (5.6%)			
Asian	4/56 (7.1%)	4/20 (20%)	0/36 (0%)			
Hispanic	1/56 (1.8%)	0/20 (0%)	1/36 (2.8%)			
Clinical Presentation & Etiology						
Etiology				**<0.001**	**<0.001**	0
SIDs	39/74 (53%)	25/25 (100%)	14/49 (29%)			
Other	35/74 (47%	0/25 (0%)	35/49 (71%)			
Chest Pain	49/61 (80%)	14/25 (56%)	35/36 (97%)	**<0.001**	**<0.001**	13
Friction Rub	16/51 (31%)	2/24 (8.3%)	14/27 (52%)	**<0.001**	**0.003**	23
Fever	29/52 (56%)	17/25 (68%)	12/27 (44%)	0.087	0.170	22
ECG suggestive of acute pericarditis	26/45 (58%)	6/19 (32%)	20/26 (77%)	**0.002**	**0.008**	29
Associated Myocarditis	10/49 (20%)	7/24 (29%)	3/25 (12%)	0.171	0.287	25
Constriction at Diagnosis	4/59 (6.8%)	2/25 (8.0%)	2/34 (5.9%)	>0.999	>0.999	15
Pericardiocentesis	12/59 (20%)	7/25 (28%)	5/34 (15%)	0.210	0.338	15
Pericardiectomy	2/59 (3.4%)	1/25 (4.0%)	1/34 (2.9%)	>0.999	>0.999	15
*Laboratory & Imaging Findings*						
Elevated CRP	60/66 (91%)	15/21 (71%)	45/45 (100%)	**<0.001**	**0.003**	8
CRP Peak Value, mg/L	62.0 (11.0, 146.0)	12.0 (6.0, 175.0)	89.5 (25.0, 146.0)	0.246	0.379	43
Troponin Positive	8/41 (20%)	4/18 (22%)	4/23 (17%)	0.713	0.912	33
LVEF Preserved	35/37 (95%)	16/17 (94%)	19/20 (95%)	0.715	0.912	37
Pericardial Effusion Present	41/46 (89%)	18/20 (90%)	23/26 (88%)	>0.999	>0.999	28
Pericardial Effusion Size	19/26 (73%)	12/17 (71%)	7/9 (78%)	0.769	0.926	48
Cardiac Tamponade	12/59 (20%)	8/25 (32%)	4/34 (12%)	0.056	0.116	15
CMR	11/45 (24%)	8/19 (42%)	3/26 (12%)	0.033	0.072	29
*Treatments*						
Prior NSAID	30/74 (41%)	5/25 (20%)	25/49 (51%)	**0.010**	**0.025**	0
Prior Colchicine	25/74 (34%)	2/25 (8.0%)	23/49 (47%)	**<0.001**	**0.003**	0
Prior Steroid	64/74 (86%)	18/25 (72%)	46/49 (94%)	0.026	0.059	0
Oral Steroid Pre-IS, mg/die	32.5 (15.0, 60.0)	60.0 (47.5, 60.0)	20.0 (10.0, 50.0)	**0.005**	**0.013**	40
IS Indication				**<0.001**	**<0.001**	0
Refractory disease	38/74 (51%)	6/25 (24%)	32/49 (65%)			
Systemic disease flare	20/74 (27%)	17/25 (68%)	3/49 (6.1%)			
Steroid-dependence	16/74 (22%)	2/25 (8.0%)	14/49 (29%)			
IS Duration, months	6.0 (2.0, 15.0)	4.5 (2.0, 15.0)	6.0 (3.0, 12.0)	0.776	0.926	35
Follow-up, months	9.5 (5.0, 21.0)	8.0 (3.0, 18.0)	12.0 (6.0, 33.0)	0.158	0.278	24
Outcomes						
Steroid Withdrawal	41/57 (72%)	9/20 (45%)	32/37 (86%)	**<0.001**	**0.003**	17
Pericarditis Resolved	69/69 (100%)	25/25 (100%)	44/44 (100%)	-	-	5
Recurrence on IS	18/72 (25%)	1/25 (4.0%)	17/47 (36%)	**0.008**	**0.003**	2

^1^ Median (Q1, Q3); n/N (%). ^2^ Wilcoxon rank sum test; Pearson’s Chi-squared test; Fisher’s exact test. ^3^ False discovery rate correction for multiple testing. Statistically significant *p*-values (*p* < 0.05) are shown in bold. *For abbreviations see Table 1*.

**Table 3:** Baseline Characteristics, Management, and Outcomes Stratified by SIDS vs. Non-SIDS Etiology.

Characteristic	Overall (N = 75) ^1^	Non-SIDs (N = 35) ^1^	SIDs (N = 40) ^1^	*p*-Value ^2^	q-Value ^3^	Missing (n)
Demographics						
Age, years	35.50 (23.00, 50.00)	30.00 (15.40, 50.00)	37.50 (26.50, 49.50)	0.225	0.407	13
Female Patient	37/62 (60%)	12/22 (55%)	25/40 (63%)	0.541	0.762	13
Ethnicity				**0.014**	**0.042**	18
African	6/57 (11%)	0/22 (0%)	6/35 (17%)			
Asian	4/57 (7.0%)	0/22 (0%)	4/35 (11%)			
Caucasian	46/57 (81%)	21/22 (95%)	25/35 (71%)			
Hispanic	1/57 (1.8%)	1/22 (4.5%)	0/35 (0%)			
Clinical Presentation & Etiology						
Autoimmune Disease	40/75 (53%)	1/35 (2.9%)	39/40 (98%)	**<0.001**	**<0.001**	0
Prior Recurrences	49/74 (66%)	35/35 (100%)	14/39 (36%)	**<0.001**	**<0.001**	1
Months Since 1st Episode	3.00 (1.00, 7.00)	3.00 (1.00, 4.00)	7.00 (1.00, 24.00)	0.570	0.773	53
Chest Pain	50/62 (81%)	22/22 (100%)	28/40 (70%)	**0.005**	**0.018**	13
Friction Rub	17/52 (33%)	11/15 (73%)	6/37 (16%)	**<0.001**	**<0.001**	23
Fever	30/53 (57%)	11/15 (73%)	19/38 (50%)	0.123	0.251	22
ECG suggestive of acute pericarditis	26/45 (58%)	12/14 (86%)	14/31 (45%)	0.011	0.034	30
*Laboratory & Imaging findings*						
LVEF Echo				>0.999	>0.999	38
Preserved	35/37 (95%)	16/16 (100%)	19/21 (90%)			
Mildly Reduced	1/37 (2.7%)	0/16 (0%)	1/21 (4.8%)			
Moderately Reduced	1/37 (2.7%)	0/16 (0%)	1/21 (4.8%)			
Pericardial Effusion	42/47 (89%)	14/15 (93%)	28/32 (88%)	>0.999	>0.999	28
Pericardial Effusion Size				0.327	0.517	48
Mild	7/27 (26%)	1/3 (33%)	6/24 (25%)			
Moderate	9/27 (33%)	2/3 (67%)	7/24 (29%)			
Severe	11/27 (41%)	0/3 (0%)	11/24 (46%)			
Cardiac Tamponade	13/60 (22%)	1/26 (3.8%)	12/34 (35%)	**0.003**	**0.014**	15
Pericardiocentesis	13/60 (22%)	2/26 (7.7%)	11/34 (32%)	0.022	0.055	15
Constriction at diagnosis	5/60 (8.3%)	0/26 (0%)	5/34 (15%)	0.063	0.141	15
Pericardiectomy	3/60 (5.0%)	0/26 (0%)	3/34 (8.8%)	0.251	0.433	15
CRP Elevated	61/67 (91%)	33/33 (100%)	28/34 (82%)	0.025	0.058	8
CRP Peak Value	68.50 (11.50, 146.00)	146.00 (146.00, 146.00)	24.00 (7.50, 106.00)	0.020	0.053	43
Troponin Positive	8/41 (20%)	2/16 (13%)	6/25 (24%)	0.448	0.654	34
Associated Myocarditis	10/49 (20%)	1/17 (5.9%)	9/32 (28%)	0.133	0.253	26
CMR	12/46 (26%)	1/20 (5.0%)	11/26 (42%)	**0.004**	**0.016**	29
Treatments						
Prior NSAID	31/75 (41%)	15/35 (43%)	16/40 (40%)	0.802	0.983	0
Prior Colchicine	26/75 (35%)	12/35 (34%)	14/40 (35%)	0.948	>0.999	0
Prior Steroid	65/75 (87%)	32/35 (91%)	33/40 (83%)	0.321	0.517	0
Oral Steroid Dose Pre-IS, mg/die	30.00 (12.50, 60.00)	40.00 (15.00, 60.00)	30.00 (12.50, 60.00)	0.708	0.928	40
First IS Drug				**<0.001**	**<0.001**	
AZA	33/75 (44%)	24/35 (69%)	9/40 (23%)			
MTX	19/75 (25%)	8/35 (23%)	11/40 (28%)			
MMF	11/75 (15%)	0	11/40 (28%)			
CYP	8/75 (11%)	1/35 (2.9%)	7/40 (18%)			
Other IS ^	4/75 (5.3%)	2/35 (5.8%)	2/40 (0.05%)			
IS Indication				**<0.001**	**<0.001**	0
Refractory-disease	39/75 (52%)	21/35 (60%)	18/40 (45%)			
Steroid-dependence	16/75 (21%)	14/35 (40%)	2/40 (5.0%)			
Systemic disease flare	20/75 (27%)	0/35 (0%)	20/40 (50%)			
Outcomes						
Steroid Withdrawal	42/58 (72%)	26/29 (90%)	16/29 (55%)	**0.003**	**0.014**	17
Recurrence on IS	19/73 (26%)	15/34 (44%)	4/39 (10%)	**0.001**	**0.005**	2

^1^ Median (Q1, Q3); n/N (%). ^2^ Wilcoxon rank sum test; Pearson’s Chi-squared test; Fisher’s exact test. ^3^ False discovery rate correction for multiple testing. Statistically significant *p*-values (*p* < 0.05) are shown in bold. *For abbreviations see Table 1*. ^ The “Other IS” category includes: Cyclosporine A (n = 1, 1.3%), Etanercept (n = 1, 1.3%), Hydroxychloroquine (n = 1, 1.3%) and Infliximab (n = 1, 1.3%).

## Data Availability

No new data were created or analyzed in this study. Data sharing is not applicable to this article.

## Supplementary Materials

The following supporting information can be downloaded at: https://www.mdpi.com/article/10.3390/medicina62050887/s1, Section S1: “Search strategy”; Figure S1: “Temporal trends in the publication of pericarditis case reports and case series stratified by underlying etiology”; Table S1: “PRISMA checklist for systematic literature review items”; Table S2: “Evaluation of methodological quality of the included case reports and case series, evaluated according to the Murad tool”. Table S3: “Baseline Demographics, Clinical Characteristics, and Treatment Outcomes of the Overall Cohort (Excluding the Aggregated Data Study [17])”; Table S4: “Baseline Characteristics, Therapeutic Management, and Clinical Outcomes. Stratified by First-Episode versus Recurrent Pericarditis (Excluding the Aggregated Data Study [17])”; Table S5: “Baseline Characteristics, Therapeutic Management, and Clinical Outcomes in SIDs vs. Non-SIDs patients (Excluding the Aggregated Data Study [17])”.
