# Supplementary material for: Expanding the Therapeutic Landscape of Pericarditis: A Systematic Review of the Use of Conventional Immunosuppressants
Source: Medicina (Kaunas). 2026 May 5;62(5):887. doi: 10.3390/medicina62050887 (PMC13208500; doi:10.3390/medicina62050887)
Supplement: Supplementary file 1 [file medicina-62-00887-s001.zip › medicina-4254161-supplementary.pdf]

SUPPLEMENTAL MATERIAL

Supplementary Material Section S1. Search strategy

The literature search was constructed utilizing a combination of controlled vocabulary (such as MeSH terms) and free-text keywords to capture two primary conceptual domains: the target condition (e.g., pericarditis and/or myopericarditis) and the therapeutic intervention (e.g., azathioprine, methotrexate, mycophenolate, and general terms for conventional immunosuppressants). Synonyms, acronyms, and related terms within each domain were grouped using the Boolean operator "OR", and the two main domains were combined using the Boolean operator "AND". This core strategy was then systematically adapted to the specific syntax and search fields (e.g., Title/Abstract/Keywords) of MEDLINE (PubMed), Embase, and Web of Science.

Supplementary Table S1. PRISMA checklist for systematic literature review items.

Section and Topic	Item #	Checklist item	Location where item is reported
**TITLE**			
Title	1	Identify the report as a systematic review.	Page 1
**ABSTRACT**			
Abstract	2	See the PRISMA 2020 for Abstracts checklist.	2
**INTRODUCTION**			
Rationale	3	Describe the rationale for the review in the context of existing knowledge.	3
Objectives	4	Provide an explicit statement of the objective(s) or question(s) the review addresses.	3
**METHODS**			
Eligibility criteria	5	Specify the inclusion and exclusion criteria for the review and how studies were grouped for the syntheses.	3-4
Information sources	6	Specify all databases, registers, websites, organisations, reference lists and other sources searched or consulted to identify studies. Specify the date when each source was last searched or consulted.	3
Search strategy	7	Present the full search strategies for all databases, registers and websites, including any filters and limits used.	Supplementary, Page 1
Selection process	8	Specify the methods used to decide whether a study met the inclusion criteria of the review, including how many reviewers screened each record and each report retrieved, whether they worked independently, and if applicable, details of automation tools used in the process.	4
Data collection process	9	Specify the methods used to collect data from reports, including how many reviewers collected data from each report, whether they worked independently, any processes for obtaining or confirming data from study investigators, and if applicable, details of automation tools used in the process.	4
Data items	10a	List and define all outcomes for which data were sought. Specify whether all results that were compatible with each outcome domain in each study were sought (e.g. for all measures, time points, analyses), and if not, the methods used to decide which results to collect.	4
	10b	List and define all other variables for which data were sought (e.g. participant and intervention characteristics, funding sources). Describe any assumptions made about any missing or unclear information.	4
Study risk of bias assessment	11	Specify the methods used to assess risk of bias in the included studies, including details of the tool(s) used, how many reviewers assessed each study and whether they worked independently, and if applicable, details of automation tools used in the process.	4 + Suppl. Table 1
Effect measures	12	Specify for each outcome the effect measure(s) (e.g. risk ratio, mean difference) used in the synthesis or presentation of results.	*Not Applicable**
Synthesis methods	13a	Describe the processes used to decide which studies were eligible for each synthesis (e.g. tabulating the study intervention characteristics and comparing against the planned groups for each synthesis (item #5)).	*N/A*
	13b	Describe any methods required to prepare the data for presentation or synthesis, such as handling of missing summary statistics, or data conversions.	*N/A*
	13c	Describe any methods used to tabulate or visually display results of individual studies and syntheses.	*N/A*
	13d	Describe any methods used to synthesize results and provide a rationale for the choice(s). If meta-analysis was performed, describe the model(s), method(s) to identify the presence and extent of statistical heterogeneity, and software package(s) used.	*N/A*

Section and Topic	Item #	Checklist item	Location where item is reported
	13e	Describe any methods used to explore possible causes of heterogeneity among study results (e.g. subgroup analysis, meta-regression).	*N/A*
	13f	Describe any sensitivity analyses conducted to assess robustness of the synthesized results.	*N/A*
Reporting bias assessment	14	Describe any methods used to assess risk of bias due to missing results in a synthesis (arising from reporting biases).	*N/A*
Certainty assessment	15	Describe any methods used to assess certainty (or confidence) in the body of evidence for an outcome.	Suppl. T1
**RESULTS**			
Study selection	16a	Describe the results of the search and selection process, from the number of records identified in the search to the number of studies included in the review, ideally using a flow diagram.	5-7 + Tables 1-3
	16b	Cite studies that might appear to meet the inclusion criteria, but which were excluded, and explain why they were excluded.	*N/A*
Study characteristics	17	Cite each included study and present its characteristics.	4, 12-15
Risk of bias in studies	18	Present assessments of risk of bias for each included study.	Suppl. T1
Results of individual studies	19	For all outcomes, present, for each study: (a) summary statistics for each group (where appropriate) and (b) an effect estimate and its precision (e.g. confidence/credible interval), ideally using structured tables or plots.	*N/A*
Results of syntheses	20a	For each synthesis, briefly summarise the characteristics and risk of bias among contributing studies.	*N/A*
	20b	Present results of all statistical syntheses conducted. If meta-analysis was done, present for each the summary estimate and its precision (e.g. confidence/credible interval) and measures of statistical heterogeneity. If comparing groups, describe the direction of the effect.	*N/A*
	20c	Present results of all investigations of possible causes of heterogeneity among study results.	*N/A*
	20d	Present results of all sensitivity analyses conducted to assess the robustness of the synthesized results.	*N/A*
Reporting biases	21	Present assessments of risk of bias due to missing results (arising from reporting biases) for each synthesis assessed.	*N/A*
Certainty of evidence	22	Present assessments of certainty (or confidence) in the body of evidence for each outcome assessed.	Suppl. T1
**DISCUSSION**			
Discussion	23a	Provide a general interpretation of the results in the context of other evidence.	8-10
	23b	Discuss any limitations of the evidence included in the review.	11
	23c	Discuss any limitations of the review processes used.	11
	23d	Discuss implications of the results for practice, policy, and future research.	8-10
**OTHER INFORMATION**			
Registration and protocol	24a	Provide registration information for the review, including register name and registration number, or state that the review was not registered.	*N/A*
	24b	Indicate where the review protocol can be accessed, or state that a protocol was not prepared.	*N/A*
	24c	Describe and explain any amendments to information provided at registration or in the protocol.	*N/A*

Section and Topic	Item #	Checklist item	Location where item is reported
Support	25	Describe sources of financial or non-financial support for the review, and the role of the funders or sponsors in the review.	*N/A*
Competing interests	26	Declare any competing interests of review authors.	*N/A*
Availability of data, code and other materials	27	Report which of the following are publicly available and where they can be found: template data collection forms; data extracted from included studies; data used for all analyses; analytic code; any other materials used in the review.	Page 1

From: Page MJ, McKenzie JE, Bossuyt PM, Boutron I, Hoffmann TC, Mulrow CD, et al. The PRISMA 2020 statement: an updated guideline for reporting systematic reviews. BMJ 2021;372:n71. doi: 10.1136/bmj.n71. This work is licensed under CC BY 4.0. To view a copy of this license, visit https://creativecommons.org/licenses/by/4.0/

*__N/A__ = **Not Applicable**. These items pertain exclusively to meta-analysis methods and results, which were not conducted in this systematic review.

Supplementary Figure S1. Temporal trends in the publication of pericarditis case reports and case series stratified by underlying etiology.

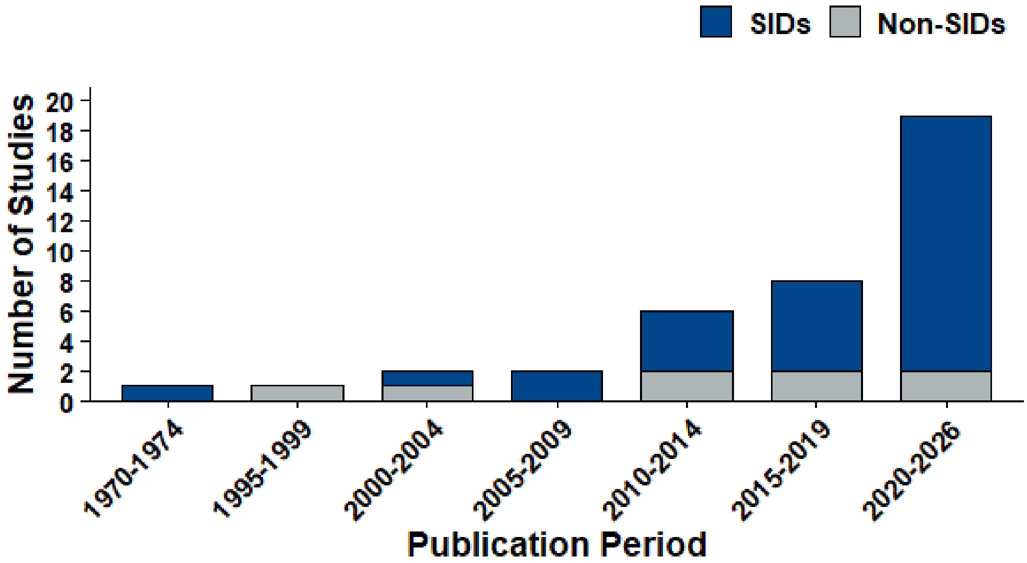

Figure Legend. Dark blue and silver bars denote cases associated with systemic immune-mediated diseases (SIDs) and non-SID etiologies, respectively. Notably, 12 of the included studies were published prior to the release of the 2015 European Society of Cardiology (ESC) guidelines on pericarditis.

Supplementary Table S2. Evaluation of methodological quality of the included case reports and case series, evaluated according to the Murad tool.

Study (Author, Year)	Selection (Q1)	Ascertainment (Q2)	Ascertainment (Q3)	Causality (Q4)	Causality (Q5)	Causality (Q6)	Causality (Q7)	Reporting (Q8)	Score
Asplen, 1970	0	1	1	1	0	0	1	1	5
Bacconnier, 2010	0	1	1	1	0	1	1	1	6
Bajraktari, 2025	0	1	1	1	0	0	0	1	4
Balak, 2011	1	1	1	1	0	1	1	1	7
Bennett, 2004	0	1	1	1	0	1	1	1	6
Bertelli, 2025	1	1	1	1	0	0	1	1	6
Boever, 2024	1	1	1	1	1	0	0	1	6
Boskovic, 2023	1	1	1	1	0	0	1	1	6
Brown, 2015	1	1	1	1	1	1	1	1	8 *(aggregated)*
Cavalcante, 2016	1	1	1	1	0	1	1	1	7
Daumas, 2011	0	1	1	1	0	0	1	1	5
Derector, 2026	0	1	1	1	0	0	1	1	5
Dey, 2019	1	1	1	1	0	1	1	1	7
Dubey, 2007	0	1	1	1	0	0	0	1	4
ElSharu, 2024	1	1	1	1	0	0	1	1	6
Fernández-Codina, 2017	0	1	1	1	1	0	1	1	6
Gomez-Alvarez, 2025	1	1	1	1	0	0	1	1	6
Higashioka, 2022	1	1	1	1	0	0	1	1	6
Horna, 2023	0	1	1	1	0	0	1	1	5
Ibrahim, 2022	0	1	1	1	1	0	1	1	6
Imadachi, 2010	0	1	1	1	0	0	0	1	4
Kato, 2021	0	1	1	1	0	0	1	1	5
Kumar, 2026	0	1	1	1	0	0	1	1	5
Lambotte, 2006	1	1	1	1	0	0	1	1	6
Luo, 2018	0	1	1	1	0	0	1	1	5
Marcolongo, 1995	1	1	1	0	1	1	1	0	6
Marijanovich, 2018	0	1	1	1	1	0	1	0	5

**Morel, 2015**	0	1	1	0	0	1	1	0	4
**Peiffer-Smadja, 2019**	1	1	1	1	0	1	1	1	7
**Pieta, 2021**	1	1	1	1	0	1	0	1	6
**Raatikka, 2003**	0	1	0	1	0	1	1	0	4
**Reynolds, 2026**	0	1	1	1	0	1	1	1	6
**Rojas-Cadena, 2025**	1	1	1	1	1	1	0	1	7
**Santos, 2023**	1	1	1	0	1	0	1	1	6
**Scott, 2011**	0	0	1	1	1	1	1	1	6
**Tahir, 2011**	0	1	1	1	0	0	1	1	5
**Tan, 2021**	1	1	1	1	0	0	1	0	5
**Thiriveedi, 2021**	0	1	1	1	0	1	1	1	6
**Verhaert, 2023**	0	1	1	1	1	1	1	1	7

Murad tool items:

#1. (*Selection*) Does the patient(s) represent(s) the whole experience of the investigator (centre) or is the selection method unclear to the extent that other patients with similar presentation may not have been reported?

#2. (*Ascertainment*) Was the exposure adequately ascertained?

#3. (*Ascertainment*) Was the outcome adequately ascertained?

#4. (*Causality*) Were other alternative causes that may explain the observation ruled out?

#5. (*Causality*) Was there a challenge/rechallenge phenomenon?

#6. (*Causality*) Was there a dose–response effect?

#7. (*Causality*) Was follow-up long enough for outcomes to occur?

#8. (*Reporting*) Is the case(s) described with sufficient details to allow other investigators to replicate the research or to allow practitioners make inferences related to their own practice?

Supplementary Table S3. Baseline Demographics, Clinical Characteristics, and Treatment Outcomes of the Overall Cohort (Excluding the Aggregated Data Study [17])

Characteristic	Overall Cohort (N=62)[1]	Missing (n)
**Demographics**		
Age, years	36.0 (23.0, 50.0)	0
Female Sex	37/62 (60%)	0
**Ethnicity**		5
African	6/57 (11%)	
Asian	4/57 (7.0%)	
Caucasian	46/57 (81%)	
Hispanic	1/57 (1.8%)	
**Pericarditis Etiology**		0
SIDS (Autoimmune)	40/62 (65%)	
Idiopathic (presumed viral)	20/62 (32%)	
Iatrogenic pericardial injury	0/62 (0%)	
Post-myocardial infarction (Dressler)	1/62 (1.6%)	
ICI-associated pericarditis	1/62 (1.6%)	
**Autoimmune Disease**	40/62 (65%)	0
Specific Autoimmune Disease		
SLE	23/42 (55%)	
RA	7/42 (17%)	
Systemic Sclerosis	2/42 (4.8%)	
EGPA	2/42 (4.8%)	
Other*	8/42 (19%)	
**Clinical Presentation**		
Prior Recurrences	36/61 (59%)	1
Months Since Previous Episode	3.0 (1.0, 7.0)	40
Chest Pain	50/62 (81%)	0
Friction Rub	17/52 (33%)	10
Fever	30/53 (57%)	9
ECG suggestive of acute pericarditis	26/45 (58%)	17
Associated myocarditis	10/36 (28%)	26
Pericardial constriction at Diagnosis	5/47 (11%)	15
Pericardiectomy	3/47 (6.4%)	15
**Laboratory & Imaging Findings**		
Elevated CRP	48/54 (89%)	8
CRP Peak Value, mg/L	69.0 (12.0, 146.0)	30
Abnormal Troponin	8/28 (29%)	34
LVEF by Echo		38
Preserved	22/24 (92%)	
Mildly Reduced	1/24 (4.2%)	
Moderately Reduced	1/24 (4.2%)	
Pericardial Effusion (present)	42/47 (89%)	15
Pericardial Effusion Size		35
Mild	7/27 (26%)	
Moderate	9/27 (33%)	
Severe	11/27 (41%)	
Cardiac Tamponade	13/47 (28%)	15
Pericardiocentesis	13/47 (28%)	15
CMR Performed	12/33 (36%)	29
**Pericarditis medical therapy**		
Prior NSAID	31/62 (50%)	0
Prior Colchicine	26/62 (42%)	0
Prior Steroid	52/62 (84%)	0
IV Steroid Dose Pre-IS, mg/day	60.0 (60.0, 250.0)	57
Oral Steroid Dose Pre-IS, mg/day	30.0 (13.0, 60.0)	27
IS therapy Indication		0
Refractory disease	39/62 (63%)	
Steroid-dependence	3/62 (4.8%)	
Systemic disease flare	20/62 (32%)	
First IS regimen		0

AZA	20/62 (32%)	
MTX	19/62 (31%)	
MMF	11/62 (18%)	
Cyc	8/62 (13%)	
Other IS regimens^	4/62 (6.5%)	
IS Duration, months	6.0 (2.0, 15.0)	23
Steroid Withdrawal	29/45 (64%)	17
Follow-up, months	10.0 (5.0, 21.0)	12
**Outcomes**		
Pericarditis Resolved	57/57 (100%)	5
Recurrence on Conventional IS	6/60 (10%)	2
Months to Recurrence	2.0 (1.5, 2.0)	59
Adverse Event	2/62 (3.2%)	0
IS Stopped for AE	2/62 (3.2%)	0

Notes: [1] Median (Q1, Q3); n/N (%). AE: adverse event; AZA: azathioprine; CMR: cardiovascular magnetic resonance; CRP: C-reactive protein; Cyc: cyclophosphamide; ECG: electrocardiogram; EGPA: eosinophilic granulomatosis with polyangiitis; ICI: immune checkpoint inhibitor; IS: immunosuppression; IV: intravenous; LVEF: left ventricular ejection fraction; MMF: mycophenolate mofetil; MTX: methotrexate; NSAID: non-steroidal anti-inflammatory drug; RA: rheumatoid arthritis; SIDS: systemic immune-mediated diseases; SLE: systemic lupus erythematosus. *Other specific autoimmune diseases include: Adult-onset Still's disease (n=2, 4.8%), Behcet's disease (n=1, 2.4%), CANDLE (n=1, 2.4%), Crohn's disease (n=1, 2.4%), Polymyositis (n=1, 2.4%), Sarcoidosis (n=1, 2.4%), and Granulomatosis with polyangiitis (GPA) (n=1, 2.4%). ^ The "Other IS" category includes: Cyclosporine A (n=1, 1.6%), Etanercept (n=1, 1.6%), Hydroxychloroquine (n=1, 1.6%) and Infliximab (n=1, 1.6%)

Supplementary Table S4. Baseline Characteristics, Therapeutic Management, and Clinical Outcomes Stratified by First-Episode versus Recurrent Pericarditis (Excluding the Aggregated Data Study [17])

Characteristic	Overall (n=61)[1]	First Event (n=25)[1]	Recurrence (n=36)[1]	p-value[2]	q-value[3]	Missing (n)
**Demographics**						
Age, years	35.0 (23.0, 49.0)	34.0 (23.0, 53.0)	37.0 (22.5, 48.0)	0.613	0.908	0
Female Sex	36/61 (59%)	12/25 (48%)	24/36 (67%)	0.145	0.322	0
Ethnicity				**0.003**	**0.009**	5
Caucasian	45/56 (80%)	12/20 (60%)	33/36 (92%)			
African	6/56 (11%)	4/20 (20%)	2/36 (5.6%)			
Asian	4/56 (7.1%)	4/20 (20%)	0/36 (0%)			
Hispanic	1/56 (1.8%)	0/20 (0%)	1/36 (2.8%)			
**Clinical Presentation & Etiology**						
Etiology				**<0.001**	**<0.001**	0
SIDs	39/61 (64%)	25/25 (100%)	14/36 (39%)			
Other	22/61 (36%)	0/25 (0%)	22/36 (61%)			
Chest Pain	49/61 (80%)	14/25 (56%)	35/36 (97%)	**<0.001**	**<0.001**	0
Friction Rub	16/51 (31%)	2/24 (8.3%)	14/27 (52%)	**<0.001**	**0.004**	10
Fever	29/52 (56%)	17/25 (68%)	12/27 (44%)	0.087	0.206	9
ECG suggestive of acute pericarditis	26/45 (58%)	6/19 (32%)	20/26 (77%)	**0.002**	**0.009**	16
Associated Myocarditis	10/36 (28%)	7/24 (29%)	3/12 (25%)	>0.999	>0.999	25
Constriction at Diagnosis	4/46 (8.7%)	2/25 (8.0%)	2/21 (9.5%)	>0.999	>0.999	15
Pericardiocentesis	12/46 (26%)	7/25 (28%)	5/21 (24%)	0.747	>0.999	15
Pericardiectomy	2/46 (4.3%)	1/25 (4.0%)	1/21 (4.8%)	>0.999	>0.999	15
**Laboratory & Imaging Findings**						
Elevated CRP	47/53 (89%)	15/21 (71%)	32/32 (100%)	**0.002**	**0.009**	8
CRP Peak Value, mg/L	62.0 (11.0, 146.0)	12.0 (6.0, 175.0)	89.5 (25.0, 146.0)	0.246	0.492	30
Troponin Positive	8/28 (29%)	4/18 (22%)	4/10 (40%)	0.400	0.696	33
LVEF Preserved	22/24 (92%)	16/17 (94%)	6/7 (86%)	0.507	0.780	37
Pericardial Effusion Present	41/46 (89%)	18/20 (90%)	23/26 (88%)	>0.999	>0.999	15
Pericardial Effusion Size*	19/26 (73%)	12/17 (71%)	7/9 (78%)	0.769	>0.999	35
Cardiac Tamponade	12/46 (26%)	8/25 (32%)	4/21 (19%)	0.319	0.608	15
CMR	11/32 (34%)	8/19 (42%)	3/13 (23%)	0.450	0.750	29
**Treatments**						
Prior NSAID	30/61 (49%)	5/25 (20%)	25/36 (69%)	**<0.001**	**<0.001**	0
Prior Colchicine	25/61 (41%)	2/25 (8.0%)	23/36 (64%)	**<0.001**	**<0.001**	0
Prior Steroid	51/61 (84%)	18/25 (72%)	33/36 (92%)	0.075	0.188	0
Oral Steroid Pre-IS, mg/die	32.5 (15.0, 60.0)	60.0 (47.5, 60.0)	20.0 (10.0, 50.0)	**0.005**	**0.015**	27
IS Indication				**<0.001**	**<0.001**	0
Refractory disease	38/61 (62%)	6/25 (24%)	32/36 (89%)			
Systemic disease flare	20/61 (33%)	17/25 (68%)	3/36 (8.3%)			
Steroid-dependence	3/61 (4.9%)	2/25 (8.0%)	1/36 (2.8%)			
IS Duration, months	6.0 (2.0, 15.0)	4.5 (2.0, 15.0)	6.0 (3.0, 12.0)	0.776	>0.999	22
Follow-up, months	9.5 (5.0, 21.0)	8.0 (3.0, 18.0)	12.0 (6.0, 33.0)	0.158	0.332	11
**Outcomes**						
Steroid Withdrawal	28/44 (64%)	9/20 (45%)	19/24 (79%)	**0.019**	0.054	17
Pericarditis Resolved	56/56 (100%)	25/25 (100%)	31/31 (100%)	-	-	5
Recurrence on IS	5/59 (8.5%)	1/25 (4.0%)	4/34 (12%)	**0.384**	0.696	2

Notes: [1] Median (Q1, Q3); n/N (%). [2] Wilcoxon rank sum test for continuous variables; Pearson's Chi-squared test or Fisher's exact test for categorical variables. [3] False discovery rate (FDR) correction for multiple testing.

Supplementary Table S5. Baseline Characteristics, Therapeutic Management, and Clinical Outcomes in SIDs vs. Non-SIDs patients (Excluding the Aggregated Data Study [17])

Characteristic	Overall (N=62)[1]	Non-SIDS (N=22)[1]	SIDS (N=40)[1]	p-value[2]	q-value[3]	Missing (n)
**Demographics**						
Age, years	35.50 (23.00, 50.00)	30.00 (15.40, 50.00)	37.50 (26.50, 49.50)	0.225	0.473	0
Female Sex	37/62 (60%)	12/22 (55%)	25/40 (63%)	0.541	0.844	0
Ethnicity				0.014	0.071	5
African	6/57 (11%)	0/22 (0%)	6/35 (17%)			
Asian	4/57 (7.0%)	0/22 (0%)	4/35 (11%)			
Caucasian	46/57 (81%)	21/22 (95%)	25/35 (71%)			
Hispanic	1/57 (1.8%)	1/22 (4.5%)	0/35 (0%)			
**Clinical Presentation & Etiology**						
Autoimmune Disease	40/62 (65%)	1/22 (4.5%)	39/40 (98%)	**<0.001**	**<0.001**	0
Prior Recurrences	36/61 (59%)	22/22 (100%)	14/39 (36%)	**<0.001**	**<0.001**	1
Months Since 1st Episode	3.00 (1.00, 7.00)	3.00 (1.00, 4.00)	7.00 (1.00, 24.00)	0.570	0.844	40
Chest Pain	50/62 (81%)	22/22 (100%)	28/40 (70%)	**0.005**	**0.034**	0
Friction Rub	17/52 (33%)	11/15 (73%)	6/37 (16%)	**<0.001**	**0.002**	10
Fever	30/53 (57%)	11/15 (73%)	19/38 (50%)	0.123	0.334	9
ECG suggestive of acute pericarditis	26/45 (58%)	12/14 (86%)	14/31 (45%)	0.011	0.062	17
**Laboratory & Imaging Findings**						
LVEF by Echo				>0.999	>0.999	38
Preserved	22/24 (92%)	3/3 (100%)	19/21 (90%)			
Mildly Reduced	1/24 (4.2%)	0/3 (0%)	1/21 (4.8%)			
Moderately Reduced	1/24 (4.2%)	0/3 (0%)	1/21 (4.8%)			
Pericardial Effusion (Present)	42/47 (89%)	14/15 (93%)	28/32 (88%)	>0.999	>0.999	15
Pericardial Effusion Size				0.327	0.594	35
Mild	7/27 (26%)	1/3 (33%)	6/24 (25%)			
Moderate	9/27 (33%)	2/3 (67%)	7/24 (29%)			
Severe	11/27 (41%)	0/3 (0%)	11/24 (46%)			
Cardiac Tamponade	13/47 (28%)	1/13 (7.7%)	12/34 (35%)	0.076	0.247	15
Pericardiocentesis	13/47 (28%)	2/13 (15%)	11/34 (32%)	0.301	0.577	15
Constriction at diagnosis	5/47 (11%)	0/13 (0%)	5/34 (15%)	0.303	0.577	15
Pericardiectomy	3/47 (6.4%)	0/13 (0%)	3/34 (8.8%)	0.550	0.844	15
Elevated CRP	48/54 (89%)	20/20 (100%)	28/34 (82%)	0.074	0.247	8
CRP Peak Value, mg/L	68.50 (11.50, 146.00)	146.00 (146.00, 146.00)	24.00 (7.50, 106.00)	0.020	0.087	30
Troponin Positive	8/28 (29%)	2/3 (67%)	6/25 (24%)	0.188	0.442	34
Associated Myocarditis	10/36 (28%)	1/4 (25%)	9/32 (28%)	>0.999	>0.999	26
CMR Performed	12/33 (36%)	1/7 (14%)	11/26 (42%)	0.223	0.473	29
**Treatments**						
Prior NSAID	31/62 (50%)	15/22 (68%)	16/40 (40%)	0.034	0.135	0
Prior Colchicine	26/62 (42%)	12/22 (55%)	14/40 (35%)	0.136	0.339	0
Prior Steroid	52/62 (84%)	19/22 (86%)	33/40 (83%)	>0.999	>0.999	0
Oral Steroid Dose Pre-IS, mg/day	30.00 (12.50, 60.00)	40.00 (15.00, 60.00)	30.00 (12.50, 60.00)	0.708	>0.999	27
First IS Drug				**0.004**	**0.034**	0
AZA	20/62 (32%)	11/22 (50%)	9/40 (23%)			
MTX	19/62 (31%)	8/22 (36%)	11/40 (28%)			
MMF	11/62 (18%)	0/22 (0%)	11/40 (28%)			
CYP	8/62 (13%)	1/22 (4.5%)	7/40 (18%)			
Other IS ^	4/62 (6.5%)	2/22 (9.1%)	2/40 (5.0%)			
IS Indication				**<0.001**	**<0.001**	0
Refractory disease	39/62 (63%)	21/22 (95%)	18/40 (45%)			
Steroid-dependence	3/62 (4.8%)	1/22 (4.5%)	2/40 (5.0%)			
Systemic disease flare	20/62 (32%)	0/22 (0%)	20/40 (50%)			

**Outcomes**						
Steroid Withdrawal	29/45 (64%)	13/16 (81%)	16/29 (55%)	0.080	0.247	17
Recurrence on IS	6/60 (10%)	2/21 (9.5%)	4/39 (10%)	>0.999	>0.999	2

Notes: [1] Median (Q1, Q3); n/N (%). [2] Wilcoxon rank sum test for continuous variables; Pearson's Chi-squared test or Fisher's exact test for categorical variables. [3] False discovery rate (FDR) correction for multiple testing. ^ The "Other IS" category includes Cyclosporine A, Etanercept, Hydroxychloroquine, and Infliximab.
